# An Electrochemical Screen-Printed Sensor Based on Gold-Nanoparticle-Decorated Reduced Graphene Oxide–Carbon Nanotubes Composites for the Determination of 17-β Estradiol

**DOI:** 10.3390/bios13040491

**Published:** 2023-04-19

**Authors:** Auwal M. Musa, Janice Kiely, Richard Luxton, Kevin C. Honeychurch

**Affiliations:** 1 Institute of Bio-Sensing Technology (IBST), University of the West of England, Bristol BS16 1QY, UK; auwal.musa@uwe.ac.uk; 2 Centre for Research in Biosciences (CRIB), School of Applied Sciences, University of the West of England, Bristol BS16 1QY, UK; janice.kiely@uwe.ac.uk (J.K.); richard.luxton@uwe.ac.uk (R.L.)

**Keywords:** estradiol, gold nanoparticles, graphene oxide, carbon nanotubes, disposable screen-printed electrodes

## Abstract

In this study, a screen-printed electrode (SPE) modified with gold-nanoparticle-decorated reduced graphene oxide–carbon nanotubes (rGO-AuNPs/CNT/SPE) was used for the determination of estradiol (E2). The AuNPs were produced through an eco-friendly method utilising plant extract, eliminating the need for severe chemicals, and remove the requirements of sophisticated fabrication methods and tedious procedures. In addition, rGO-AuNP serves as a dispersant for the CNT to improve the dispersion stability of CNTs. The composite material, rGO-AuNPs/CNT, underwent characterisation through scanning electron microscopy (SEM), ultraviolet–visible absorption spectroscopy (UV–vis), Fourier-transform infrared (FTIR) spectroscopy, and atomic force microscopy (AFM). The electrochemical performance of the modified SPE for estradiol oxidation was characterised using cyclic voltammetry (CV) and differential pulse voltammetry (DPV) techniques. The rGO-AuNPs/CNT/SPE exhibited a notable improvement compared to bare/SPE and GO-CNT/SPE, as evidenced by the relative peak currents. Additionally, we employed a baseline correction algorithm to accurately adjust the sensor response while eliminating extraneous background components that are typically present in voltammetric experiments. The optimised estradiol sensor offers linear sensitivity from 0.05–1.00 µM, with a detection limit of 3 nM based on three times the standard deviation (3δ). Notably, this sensing approach yields stable, repeatable, and reproducible outcomes. Assessment of drinking water samples indicated an average recovery rate of 97.5% for samples enriched with E2 at concentrations as low as 0.5 µM%, accompanied by only a modest coefficient of variation (%CV) value of 2.7%.

## 1. Introduction

In recent years, there has been growing concern over the presence of endocrine-disrupting chemicals in the environment. These substances have the potential to impede growth and development across a wide array of species [1], underscoring the importance of developing methods that can detect their occurrence [2]. Among these pollutants, 17-β-estradiol (E2) is particularly worrisome due to its reported ability as a steroidal oestrogen to disrupt endocrine function by interfering with oestrogen receptors, leading to reproductive system abnormalities and immune dysfunction, among other effects [3,4]. The need for rapid, economic, reproducible, sensitive, and accurate detection methods is apparent; however, most currently available techniques require specialised personnel and access to well-equipped laboratories [5], making them relatively costly. Electrochemical sensors are a versatile and advantageous analytical tool, providing various benefits such as affordability, high sensitivity, portability, and user-friendliness. All that is required for their optimal operation is a low-cost potentiostat. These advanced sensors can be modified to provide dynamic ranges across varying concentrations, from extremely low levels to parts per million (ppm) or higher, indicating their adaptability in diverse settings. Electroanalytical methods have emerged over the past few decades as effective alternatives to traditional analysis techniques, due to their unique capabilities in accumulating various analytes on electrode surfaces while eliminating associated drawbacks [4]. Among these methods, disposable screen-printed electrodes (SPEs) have been notably recognised for their utility and practicality. They promise alternatives to the common three-electrode setup in their portability, measurement speed, mass production, and low cost [5,6]. However, some of the components within the ink formulation of a screen-printed electrode are non-conductive, resulting in poor conductivity and performance, ultimately affecting the performance of the SPE.

Screen-printed electrodes (SPEs) present a promising option for replacing the typical three-electrode configuration, due to their portability, rapid measurement capabilities, low cost, and potential for mass production [5,6]. Nevertheless, some non-conductive constituents make up the ink formulation of an SPE, which can impede its conductivity and overall effectiveness, consequently compromising its performance. Therefore, there is a need to improve the conductivity of SPEs by either modifying their ink formulation or employing additional conductive materials to enhance the electrode’s performance.

Several studies [7,8,9,10,11,12,13,14,15,16,17,18] have reported the determination of E2 through different sensor surfaces. Electrochemical oxidation of the phenolic group present in the hormone leads to electrode fouling, due to the formation of an insoluble layer on its surface, resulting in electrode passivation and negatively impacting the analytical performance [19]. Hence, it is necessary to explore new materials for SPEs’ modification that can enhance their analytical capabilities [20].

Electrochemical sensors incorporating carbon-based nanomaterials have been employed to detect various analytes [21]. Among these, graphene (Gr) and carbon nanotubes (CNTs) are frequently used in sensor fabrication, owing to their significant electrochemical windows, high conductivity, large specific surface area, and stability that enhance electron transfer reactions [21,22,23] while also promoting electrochemical reactivity [24]. Additionally, research indicates that fouling is less likely with graphene [25], further establishing its potential as a suitable electrode modification material alongside CNTs [26,27,28,29,30,31,32,33,34,35,36,37].

Gold nanoparticles (AuNPs) have been widely employed in the development of electrochemical sensors [38,39,40,41] through various synthesis methods, including physical, chemical, biological, and electrochemical techniques [38]. The use of plant extracts as bio-reductants for producing metallic nanoparticles has received considerable attention in research by previous reports [42,43]. This approach obviates the necessity for noxious chemicals that can be corrosive or harmful and advocates a sustainable methodology for their production. A variety of plant extracts—such as lemongrass (*Cymbopogon citratus*), *Zingiber oicinale*, *Acalypha indica*, and *Abelmoschus esculentus*—have been utilised for the preparation of AuNPs. In addition to electrochemical reduction methods, thermal and photocatalytic reduction methods have also been employed in this context [44]. Notably, bay leaf extract from *laurus nobilis* (bay laurel) has been reported as a promising alternative for generating AuNPs without the requirement for stabilising or capping agents. Moreover, it has demonstrated efficacy in reducing graphene oxide, serving a dual purpose [45,46].

The combination of graphene and CNTs as a composite has been extensively researched for various electrochemical sensor applications [27]. Carbon nanomaterials, including CNTs and graphene, have shown promise in immobilising metal nanoparticles [47], enhancing their individual properties through material synergy. This ultimately leads to improved electrochemical sensitivity within sensors [48]. It is necessary to disperse these materials using solvents and surfactants before electrode modification can occur with CNTs and graphene. However, the abundant oxygen within graphene oxide allows it to serve as a dispersant agent for CNTs, improving the overall composite properties and preventing irreversible aggregation of the graphene material. A careful approach towards tailoring these materials will prove promising during efforts towards the development of novel hybrid nanocomposites [29].

This research investigates an eco-friendly method for fabricating a composite material based on reduced graphene oxide decorated with gold nanoparticles synthesized via a sustainable, single-step method without the requirement of intricate processes or harsh chemicals. This approach involves employing plant extracts to synthesize AuNPs and bio-reducing graphene oxide in conjunction with rGO acting as a dispersant for CNTs. Additionally, this methodology adheres to non-toxic standards aligned with the 12 principles of green chemistry when producing an electrochemical sensor [49,50].

## 2. Materials and Methods

### 2.1. Chemicals and Reagents

Bay laurel leaves were purchased from JustIngredients Ltd. (UK). Graphene oxide with 4–10% edge-oxidised and carboxylic-acid-functionalised multiwalled carbon nanotubes (>8%), with an average diameter of 9.5 nm and a length of 1.5 μm, was used in the study. Chemicals such as gold chloride trihydrate HAuCl_4_·3H_2_O, sodium hydroxide NaOH, potassium ferricyanide K_3_[Fe(CN)_6_], potassium ferrocyanide K_4_[Fe(CN)_6_], potassium chloride KCl, glacial acetic acid, phosphoric acid H_3_PO_4_, boric acid H_3_BO_3_, and sulfuric acid H_2_SO_4_, along with gold(II) chloride and estradiol standard, were obtained from Merck (Dorset, UK). A solution of E2 (0.01 M), prepared in ethanol, was stored at +4 °C for future use. Deionised water from Suez Water System (UK) was used to prepare solutions for the study. A potassium ferricyanide solution was created by dissolving K_3_[Fe(CN)_6_] and potassium ferrocyanide K_4_[Fe(CN)_6_] in 0.1 M KCl and deionised water at concentrations of 5 mM, respectively. Britton–Robinson buffer solution (B-R) with varying pH levels between 2 and 8 was made using acetic acid (0.04 M), boric acid (0.04 M), phosphoric acid (0.04 M), and NaOH as needed to adjust the pH levels accordingly.

### 2.2. Apparatus and Instrumentation 

All voltammetric measurements were carried out using a PalmSens EmStat3 Potentiostat (PalmSens BV, Houten, the Netherlands) and PSTrace 5.8 software for instrument control and data acquisition during the voltammetric measurements. Screen-printed carbon electrodes (SPEs), obtained from Palintest Limited (Gateshead, UK), were used in a three-electrode system that included a working electrode, a carbon counter-electrode, and a silver reference electrode. The working electrode was modified in some cases. The materials were characterised using scanning electron microscopy (Oxford Instruments SEM, 20 kV) and energy-dispersive X-ray spectroscopy (EDX) with an Oxford Instruments AZtec E.D.S. Atomic force microscopy (AFM) was conducted with a Bruker-Innova AFM instrument. Fourier-transform infrared spectroscopy using attenuated total reflection (ATR-FTIR) was carried out on a PerkinElmer Spectrum 1000 spectrometer. An Agilent Cary 60 UV–VIS spectrophotometer in Santa Clara, United States, was used for ultraviolet–visible absorption spectroscopic measurements.

Cyclic voltammograms were initially recorded with blank buffer solutions, and then in the same solutions containing E2. A starting potential of 0.0 V, an initial switching potential of 0.8 V, and an end potential of 0.0 V were utilised. Differential pulse voltammetry was undertaken using a starting potential of 0.0 V and a final potential of 0.8 V, using a step height of 7 mV, pulse repetition time of 0.5 s, and pulse amplitude of 100 mV. The DPV parameters were selected based on previous works reported in the literature for estradiol sensing [51].

#### Synthesis of rGO-AuNPs/CNT and Electrode Fabrication

Gold-nanoparticle-decorated reduced graphene/carbon nanotubes composites were prepared by methods adapted from our group, with a few modifications [52]. Briefly, bay leaf extract was prepared by grinding dried bay leaves to a powder, adding the powdered leaves to deionised water at 80 °C for 10 min, and then straining and centrifuging the resultant solution to remove plant material. The bay leaf extract was then stored at 4 °C and used within four weeks. Equal volumes of the abovementioned GO suspension, bay leaf extract, and HAuCl_4_ (10 mM) (1:1:1) *v*/*v*/ were then mixed and kept overnight for nanoparticle formation, where the light-yellow-coloured mixture changed to a wine-red colour, indicating the formation of AuNPs [53]. The decorated graphene oxide containing AuNPs was extracted by centrifugation at 5000 rpm for 15 min, washed three times in deionised water, and then suspended again in deionised water with the concentration of GO set to 0.05 mg/mL. Carbon nanotubes (0.1 mg/mL) were added to the rGO-AuNPs and then ultrasonicated for three hours to ensure full dispersion of the rGO-AuNPs/CNT. Finally, the SPE was modified with rGO-AuNPs/CNT by drop-casting suspensions of either 0.3, 0.5, 0.7, or 1.0 µL on the working electrode of the SPE. These were then allowed to dry at room temperature. An untreated MWCNT/GO concentration dispersion was also created. AuNP solutions without GO or MWCNTs were prepared for characterisation as previously stated.

**Figure 1:**
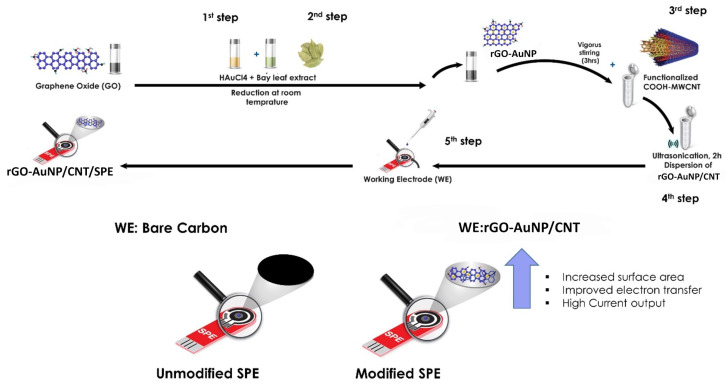
Scheme for the rGO-AuNP/CNT modified screen-printed electrode’s fabrication process.

A graphical representation of the steps of modification of the rGO-AuNP/SPE sensor’s fabrication is shown in Figure 1.

## 3. Results

### 3.1. Characterisation of rGO-AuNPs/CNT

**Figure 2:**
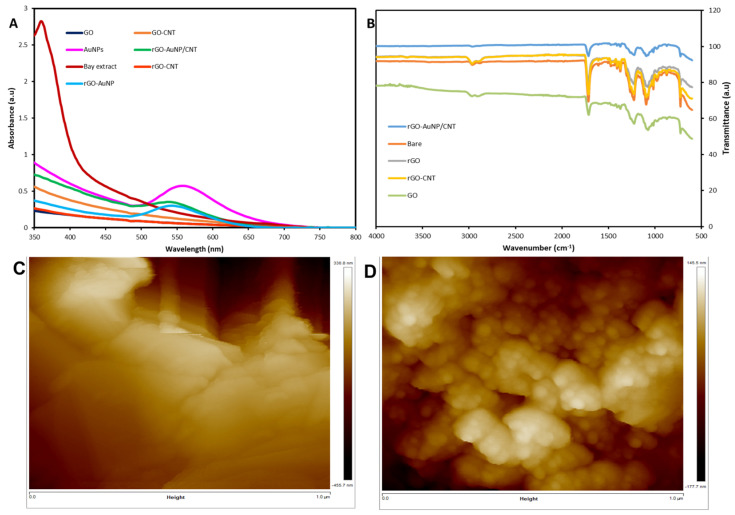
(**A**) UV−vis absorbance spectra of GO, AuNPs, bay laurel extract, rGO-AuNPs, GO-CNT, rGO-CNT, and rGO-AuNPs/CNT. (**B**) FTIR spectra of rGO-AuNPs/CNT/SPE, rGO, rGO-CNT, GO and bare SPE). (**C**) AFM images of bare SPE and (**D**) rGO-AuNPs/CNT/SPE as model electrodes.

In Figure 2, the UV–Vis spectra of various substances—including bay leaf extract, rGO-AuNPs/CNT, AuNPs, rGO-AuNP, rGO-CNT, GO/CNT, and GO—are depicted. The absorption peak at 560 nm observed in the spectrum of the AuNPs is indicative of successful formation using bay leaf extract, since this falls within a typical range for such particles. Additionally, a similar peak was discerned in rGO-AuNPs/CNT, implying that these composites also contain AuNPs. Previous studies have reported absorbance levels for AuNPs between 500 and 550 nm [42,53]. The rGO-AuNPs/CNT shows a broad absorbance peak around a wavelength of 560 nm, indicating the presence of AuNPs. The absorption peaks observed here correspond to the wavelengths given in the literature for AuNPs at 520, 522, 524, 528, and 530 nm, respectively [42,53], and are consistent with the typically quoted absorption seen at 500–550 nm for AuNPs. The difference in peak values (absorption) compared to the reports in the literature can be attributed to the presence of GO in the solution. No peaks were seen for GO, rGO-CNT, GO-CNT, and bay leaf extract. Wang et al. reported the GO spectrum peak at 226 nm [54]. A shoulder at ∼300 nm can be attributed to the n→π* transition of the carbonyl groups (C=O bond), similar to a previous report [55]. However, this study was carried out between 400 and 800 nm, which would explain why no GO peaks were recorded. These absorption peaks cannot be seen in the spectrum of rGO-AuNPs/CNT, indicating that these free carboxyl groups are committed to accommodating AuNPs.

The FTIR spectrum of the bare SPE (illustrated by the orange spectrum in Figure 2B) demonstrates distinct peaks at 2966 cm^−1^, 1714 cm^−1^, 1227 cm^−1^, and 1094 cm^−1^ that correspond to OH, CH_3_, COOCH_3_, and C=O functional groups, respectively. These same groups were also identified not as well-defined peaks in the rGO-CNT/AuNPs (depicted by the blue spectrum in Figure 2B), indicating that oxygen functionalities had been eliminated during reduction. A similar occurrence has been reported with plant extracts converted graphene nanosheets (PCGN) [56].

Furthermore, we examined the morphological characterisation in terms of surface topography and roughness [46,57] of the rGO-AuNPs/CNT using atomic force microscopy (AFM). The respective AFM images of bare and rGO-AuNP/CNT SPE’s in Figure 2C,D displays morphological AFM images of the rGO-AuNPs/CNT composite material, indicating a thick compacted uniform sheet of material [56]. The surface of the rGO-AuNP/CNT (Figure 2D) showed a significant increase in surface roughness compared with that of the bare SPE (Figure 2C). It is noteworthy that the drop-casting procedure can yield agglomerations of material [56]. In Figure 2C, clear rough surfaces with sharp layered steps and terraces can be observed in the topography of the carbon ink on the bare SPE. No peak-to-peak interlayer distance was measured, due to the lack of X-ray diffraction measurements of the composite. 

**Figure 3:**
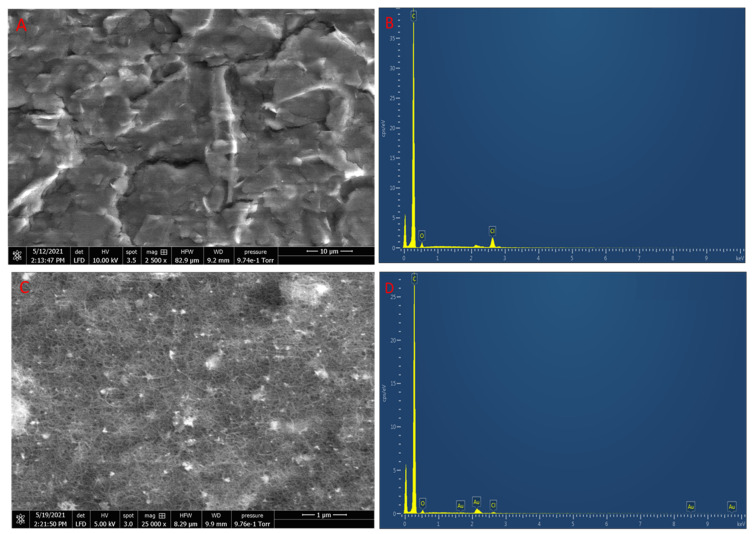
(**A**) SEM image of bare SPE. (**B**) EDS image of bare SPE showing elemental C, O, and Cl. (**C**) SEM image of rGO-AuNP/CNT/SPE at 25,000× magnification, with no observable rGO. (**D**) EDS image of rGO-AuNPs/CNT/SPE showing elemental C, O, Cl, and Au.

Figure 3A–D show SEM images of the bare SPE and the rGO-AuNPs/CNT/SPE. As can be seen in Figure 3A, the bare SPE shows graphitic sheet-like structures as typical of the SPE surface. According to the composition of the electrode, these structures can be assigned as graphitic carbon powder. SPEs have been reported to show small particles dispersed throughout the surface electrodes based on the different curing temperatures suggested by [58,59] and the composition of the ink. These structures can be assigned as graphitic graphite particles illustrating typical features of a non-uniform pattern from different graphite particles. Figure 3C shows uniform mesh-like structures that result from the presence of rGO-AuNPs/CNT, which covered and bridged the graphene sheet and small particles of AuNPs. Graphene oxide has been reported to have individual sheets formed in bundles, while reduced graphene has flocculent flake-type layers [60]; however, the presence of CNTs alters the behaviour of graphene, as illustrated in the figure, showing a CNT-interwoven mesh of nanotubes, with thin layers of space between each nanotube [61]. This can be attributed to reducing the graphene oxide to sheet-like graphene with ‘sprinkled’ AuNPs. Figure 3C shows the rGO-AuNPs/CNT/SPE, depicting the presence of well-decorated graphene oxide–carbon nanotubes. The synthesis of gold nanoparticles via this route has the following advantages: (a) applying a bio-reduction synthesis route without using a hazardous reducing agent such as sodium borohydride; (b) AuNPs can be attached to the surface more accessible to the rGO/CNT surface to form a composite [62,63].

To confirm the assembly of AuNPs and the surface composition of rGO-AuNPs/CNT, EDS analysis was performed. Figure 3D shows the resulting EDS spectra. This suggests that the composite mainly includes the elements C, O, Au, and Cl. The observed Au elemental peaks are indicative of AuNPs being incorporated into the composite, indicating the successful incorporation of the AuNPs—unlike the bare unmodified SPE, which was characterised by C, O, and Cl (Figure 3B). We speculated that the presence of Cl in this spectrum could have stemmed from the ink itself, as seen in the bare SPE. A similar phenomenon has been reported in electrochemically synthesised AuNPs/single-walled carbon nanotube hybrids [64]. This study revealed that the bare SPE is not comparable with the rGO-AuNPs/CNT material, which is obviously smoother compared to the bare SPE, which appeared clumped and stacked [64]. Jian et al. reported similar results for the electrochemically reduced graphene oxide/AuNPs [58,65,66].

### 3.2. Electrochemical Characterisation

**Figure 4:**
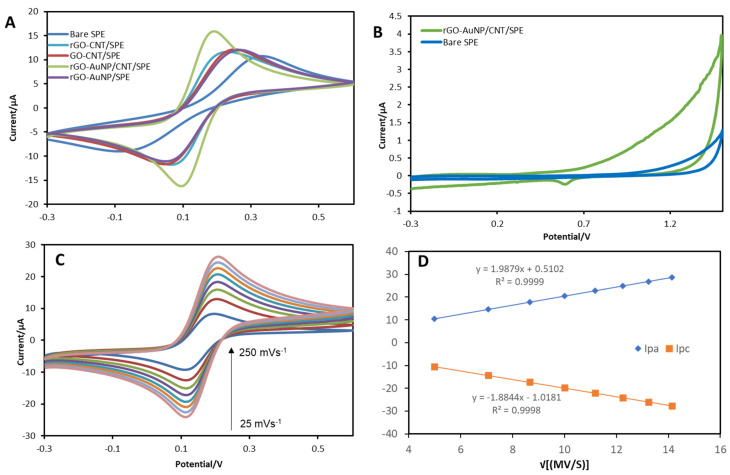
(**A**) CV of (a) bare SPE, rGO-CNT/SPE, GO-CNT/SPE, rGO-AuNPs/CNT/SPE, and rGO-AuNP immersed in 0.1 M KCl containing 5 mM [Fe(CN)_6_]^−3/−4^. (**B**) Cyclic voltammograms of bare SPE (blue) and rGO-AuNPs-CNT/SPE (green) in a 0.5 M H_2_SO_4_ solution. Scan rate: 50 mVs^−1^. At 100 mVs^−1^ scan rate: (**C**) CVs of rGO-AuNPs/CNT/SPE at different scan rates (25–250 mVs^−1^) in 0.1 M KCl containing 5 mM [Fe(CN)_6_]^−3/−4^. (**D**) Plots of anodic current peak vs. square root of scan rate and cathodic current peak vs. square root of scan rate.

In Figure 4A, the CVs of bare SPE, rGO-CNT/SPE, GO-CNT/SPE, rGO-AuNPs/CNT/SPE, and rGO-AuNP are illustrated immersed in 0.1 M KCl containing a mixture of ferricyanide/ferrocyanide at concentrations of 5 mM [Fe(CN)_6_]^−3/−4^at 100 mVs^−1^. According to Figure 4A, the peak current of redox was consistently for GO-CNT, rGO-AuNP, and rGO-CNT and remained unaffected by any discernible impact observed from rGO-AuNPs/CNT. Hence, it was concluded that model electrodes in subsequent measurements would use rGO-AuNPs/CNT, GO-CNT benchmarked to bare SPE. Further characterisation can be found provided in Supplementary Information (S).

In Figure 4B, the cyclic voltammograms for bare SPE and rGO-AuNPs/CNT/SPEs are displayed in solution containing 0.5 M H_2_SO_4_ under the same experimental conditions are presented. The data show that at a scan rate of 50 mV/s and in the solution containing 0.5 M H_2_SO_4_, a single reduction peak of oxide species was observed on the rGO-AuNPs/CNT/SPE electrode, at around 0.58 V, corresponding to gold oxide reduction, indicating successful voltammetric reduction of gold oxide by this material. This result is consistent with previous findings [66] documenting Au surface behaviour during sulfuric acid exposure [63,64,67,68,69]. 

In Figure 4C, the CVs of an rGO-AuNPs/CNT/SPE at different scan rates (ranging from 25 to 250 mVs^−1^), with the same concentration and solution as above, are presented. Linear correlations between the anodic/cathodic current peak and the square root of the scan rate were observed over the range examined, as can be seen in Figure 4D.

The utilisation of the rGO-AuNPs/CNT/SPE composite led to an increase in the specific surface area, resulting in well-defined cathodic and anodic peak currents. The modification also improved the ΔEp separation by 91.94 mV as compared to the bare SPE, which had a value of 331.8 mV, suggesting a faster electron transfer processes. This heightened efficiency could be attributed to the rGO-AuNPs/CNT modifier facilitating charge transfer for [Fe(CN)_6_]^−3/−4^ through improvements in conductivity, while overcoming potential hindrances caused by insulating layers, according to previous research [70].

The rGO-AuNPs/CNT/SPE composite exhibited an improved electrochemical response due to the presence of AuNPs, which could be attributed to their large surface-to-volume ratio, superior catalytic activity, and enhanced electrical conductivity. This finding corroborates Wang et al.’s research on an rGO/CNT/AuNPs-SPE, where a peak-to-peak separation of 97 mV was achieved for Fe(CN)_6_ detection using an electrochemical sensor [70]. In Figure 4D, a linear correlation between the anodic and cathodic peak currents implies that the uniform deposition of the composite’s electroactive thin layer contributes significantly to its overall electrochemical behaviour [71]. The peak potential shift indicates slow electron transfer and a quasi-reversible reaction for the unmodified SPE. In addition, the theoretically ideal equal number of electron oxidation/reduction reactions due to this shift would not be observed.

We next investigated the electrochemically active surface area of the electrode, which are essential factors in electrochemical processes occurring at the electrode surface in electrochemical sensors [72,73]. The electrochemical active surface area of the modified electrode was measured using 5 mM [Fe(CN)_6_]^−3/−4^ as the redox probe. The current for the electrochemical reaction of ferrocyanide (at a mass-transfer-limited rate) that diffuses to an electrode surface is described by the Randles–Sevcik equation [72].

The electrochemical active surface area of rGO-AuNPs/CNT was calculated using Equation (1) for the peak current (Ip) [74]:

*I_p_* = (2.69 × 10^5^) *AD*^1/2^
*n*^3⁄2^ *v*^1⁄2^*C* (1)

where *n* is the number of electrons, *A* is the electroactive surface area of the rGO-AuNPs/CNT/SPE (cm^2^), *D* is the diffusion coefficient of the redox marker [Fe(CN)_6_]^−3/−4^ (*D* = 7.2 × 10^−6^ cm^2^ s^−1^ [74,75] in 0.1 M KCl), *C* is the concentration of the redox probe in the solution (M), and v is the scan rate (V/s). Figure 4A shows an increase in the peak currents for the [Fe(CN)_6_]^4−/3−^ redox couple at the rGO-AuNPs/CNT/SPE compared to that at the bare, unmodified SPE. These results indicate that the electron transfer is enhanced by modifying the bare SPE with rGO-AuNPs/CNT. The surface area value was estimated to be 0.014 cm^2^ for the rGO-AuNPs/CNT/SPE–significantly higher than the bare SPE’s figure of 0.009 cm^2^. Scan rate studies were carried out using equimolar ferri/ferrocyanide at 5 mM [Fe(CN)_6_]^−3/4−^. Both showed a linear relationship between peak current and the square root of the scan rate, indicating a diffusion-limited response in all cases. The electroactive surface area values of all SPEs are provided in Supplementary Information (Table S1).

#### Voltammetric Behaviour of 17β-Estradiol

**Figure 5:**
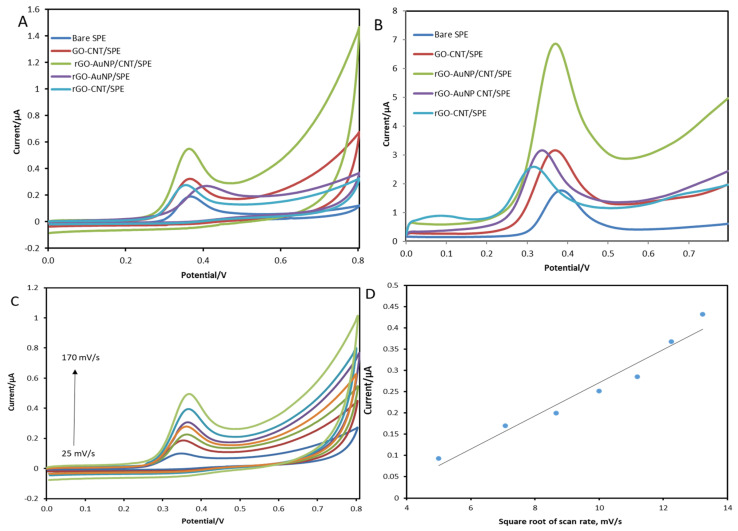
(**A**) Cyclic voltammograms (CVs) of the bare SPE, GO-CNT/SPE, rGO-AuNPs/CNT/SPE, rGO-AuNPs/SPE, and rGO-CNT/SPE in 20 µM E2 at 100 mVs^−1^. (**B**) Differential pulse voltammograms (DPVs) of the bare SPE, GO-CNT/SPE, rGO-AuNPs/CNT/SPE, rGO-AuNPs/SPE, and rGO-CNT/SPE in 20 µM E2 in Britton–Robinson buffer (pH 5). (**C**) Cyclic voltammograms (CVs) of rGO-AuNPs/CNT/SPE in 20 µM E2 at a 25–170 mVs^−1^ scan rate. (**D**) Plot of current peak vs. square root of scan rate.

The modified SPEs were utilised to study the electro-oxidation of E2, as depicted in Figure 5. The CVs for the bare SPE, rGO-CNT/SPE, GO-CNT/SPE, rGO-AuNPs/CNT/SPE, and rGO-AuNP were recorded at pH 5 using a B-R buffer solution with a 20 μM concentration of E2. To achieve greater depth, an investigation was conducted into the influence of various configurations of modified SPEs—on the oxidation peaks of the estradiol, as shown in Figure 5A,B. Figure 5A revealed shows varying oxidation peaks obtained, according to the Figure 5A below with rGO-AuNPs/CNT had the highest, followed by GO-CNT. Also, the differential pulse voltammograms of the bare SPE, GO-CNT/SPE, and rGO-AuNPs/CNT/SPE at 20 μM E2 are depicted in Figure 5B. The results show that the E2 peak current for the bare SPE, GO/CNT/SPE, and rGO-AuNPs/CNT/SPE is elevated compared to CV. This is because differential pulse voltammetry (DPV) is a frequently used electrochemical technique due to its heightened sensitivity and specificity in detecting different analytes. This enables better differentiation between charging and Faradaic currents [16]. The oxidation peak current recorded at +0.385 V was 1.48 µA for the bare SPE, while GO-CNT/SPE exhibited a value of 2.95 µA at 0.392 V, and rGO-AuNPs/CNT/SPE showed an even higher reading of 5.079 µA at 0.371 V.

Meanwhile, rGO-AuNPs and rGO-CNT had no discernible impact on estradiol’s peak current, which maintained a consistent value of approximately 2.9 µA (as depicted in Figure 5B). As such, it was determined that rGO-AuNPs/CNT would be utilised for subsequent measurements against the bare SPE as model electrodes.

The outcome revealed that the rGO-AuNPs/CNT/SPE had the highest response for E2 peak current, as compared to the GO-CNT/SPE and bare electrodes. At + 0.363 V, an oxidation peak current value of 0.19 µA was observed on the unmodified SPE surface, whereas it was found to be higher (i.e., 0.322 µA at 0.359 V and 0.536 µA at + 0.363 V) for the GO-CNT/SPE and rGO-AuNPs/CNT/SPE surfaces, respectively; this is consistent with the prior literature [76]. This increase was due to the rGO-AuNPs/CNT material properties providing enhanced electron transfer.

Moreover, to clarify the oxidation mechanism of E2 at the rGO-AuNPs/CNT electrode in a Britton–Robinson solution with pH 5, cyclic voltammetry studies were conducted at varying scan rates between 25 and 170 mVs^−1^ (Figure 5C). The peak currents obtained demonstrated a linear correlation with the square root of scan rate, indicating that the process is controlled by diffusion (Figure 5D).

### 3.3. Effect of pH

**Figure 6:**
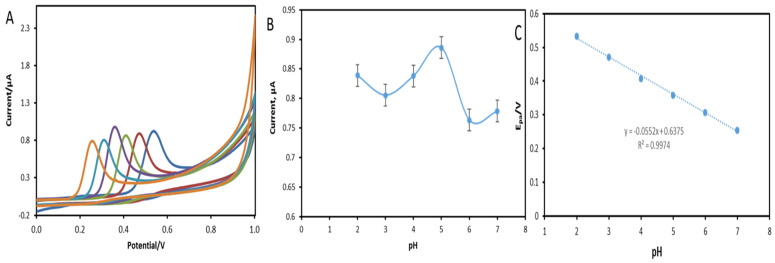
(**A**) CVs of the effects of pH on the oxidation. (**B**) Plot of estradiol peak current vs. pH. (**C**) A plot of peak potential versus pH.

Figure 6 shows the effects of pH on the direct oxidation of E2 at the rGO-AuNPs/CNT electrode. Values of pH ranging from 2 to 7 were investigated, with rGO-AuNPs/CNT having the highest peak currents (Ip) at pH 5 (Figure 6C). According to the equation, the E2 peak potential would move to more negative potential values with increasing pH (Ep = −0.0552, pH + 0.6375, indicating a 55 mV/pH slope). This slope is close to the Nernst theoretical value of 59 mV/pH for an oxidation involving an equal number of electrons and protons. Consequently, a pH 5 0.04 M Britton–Robison buffer was selected for subsequent studies. Various electrolytes have been studied for the oxidation of E2 as shown in the Supplementary Information in Figure S1. A similar pH value was the choice for optimum estradiol determination using voltammetry by Ozcan et al. [77]. Based on this information, and result below, pH 5 was selected for subsequent experiments.

### 3.4. Optimisation of Modifier

**Figure 7:**
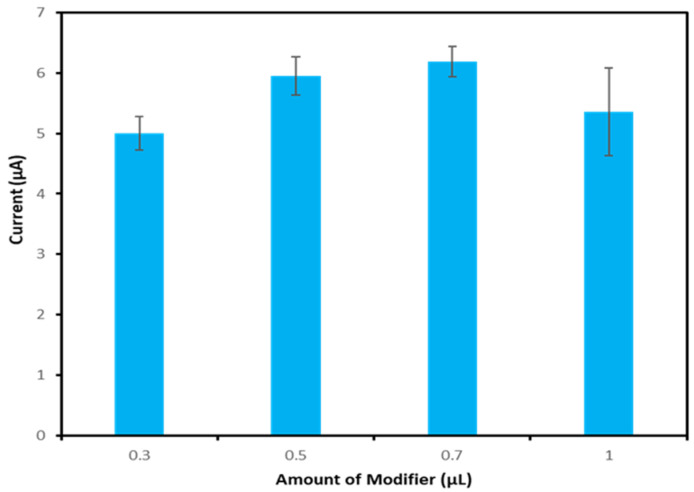
Bar chart of DPV oxidation peak current of E2 vs. the amount of rGO-AuNPs/CNT modifier.

Figure 7 shows the effects of utilising different amounts of rGO-AuNPs/CNT to modify the SPE in terms of its resulting sensitivity. Volumes of 0.3–1.0 µL of the rGO-AuNPs/CNT suspension were drop-casted onto the working electrode. Figure 5 shows the effects of the composites on the direct oxidation of E2 on the rGO-AuNPs/CNT electrode. The highest peak current was obtained for 0.7 µL of suspension; consequently, this volume was selected to modify the SPE in further investigations. As the amount of the rGO-AuNPs/CNT modifier increased, the peak current decreased. A further study that was carried out involved adding 0.7 µL of the modifier in the form of layer-by-layer modification (2x). However, the resulting current was observed to decrease using this approach, which was considered to be due to the aggregation of the modifier particles and the stacking of various materials in the composite. Consequently, a single 0.7 µL drop-cast was utilised for the rest of the experiments.

### 3.5. Chemometric Fourier Analysis

The discipline of chemometrics has found utility in the realm of electrochemistry for differentiating faradaic current contributions from background interferences arising due to charging current. A multitude of approaches have been employed, including curve-fitting [78], Kalman filters [79], derivative techniques [80], and Fourier transforms [72,81,82]. Carvalho et al. implemented Fourier-transform-based noise reduction methods in voltammetric analysis to minimise background interference effects [83]. This study used a multivariate calibration method to replicate and evaluate voltammetric data [83]. Additionally, Górski et al. [84] created an algorithm that addresses the contribution of net faradaic current. The report indicated that implementing the algorithm effectively reduced the background current and enhanced the signal-to-noise ratio [84]. Consequently, DPV can be regarded as an efficient electroanalytical technique with established benefits, such as superior discrimination against background currents and low detection or determination limits when combined with appropriate data processing algorithms. This led to exploring rGO-AuNPs/CNT/SPE analytical capabilities for detecting E2 using DPV.

### 3.6. Calibration Curve and Limit of Detection

**Figure 8:**
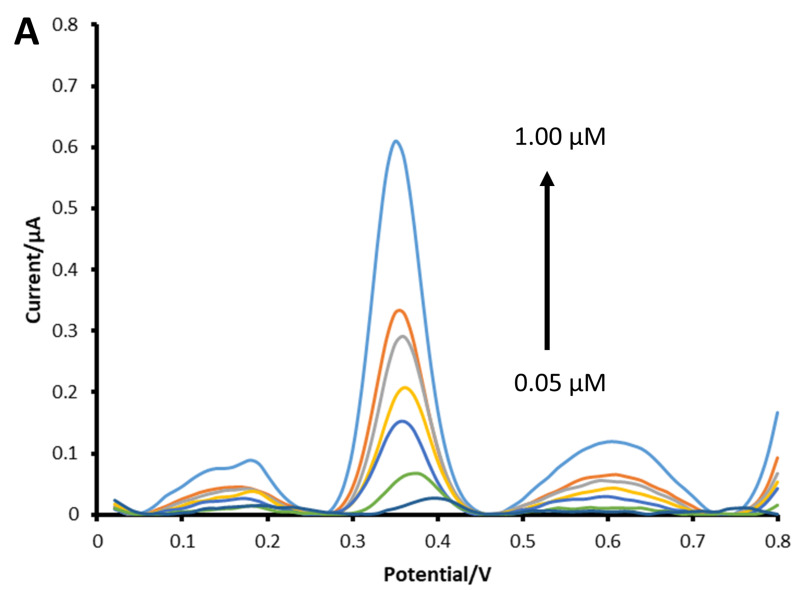

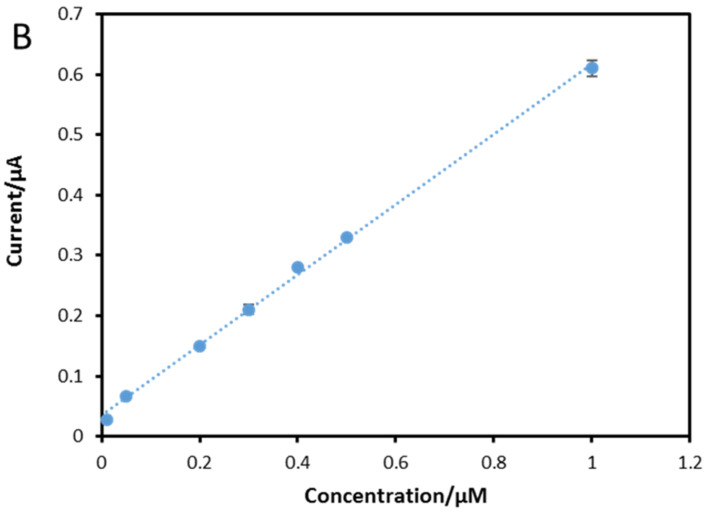
(**A**) DPVs of RGO-AuNPs/CNT/SPEs with different concentrations of E2 (0.05–1 µM) in B-R buffer (pH 5). Automatic baseline correction was carried out based on the report of Górski et al. [84]. (**B**) The oxidation peak current plot against the estradiol concentration, with *y* = 0.583x and + 0.0343 *R^2^* = 0.9982. Error bar: standard deviation for *n* = 3.

To examine the sensor’s response to various concentrations of E2 (ranging from 0.05 to 1.0 µM), differential pulse voltammetry was performed utilising a modified SPE consisting of rGO-AuNPs/CNT. Calibration plots were generated by analysing the peak current, and each investigation was repeated three times to ensure accuracy. The limits of detection (LOD) and quantification (LOQ) were computed as LOD = 3 δ/m and LOQ = 10 δ/m, respectively, with δ being the standard deviation for oxidation peak currents at the lowest detectable concentration, while m represents the slope gradient in the calibration curve analysis. This method enabled the accurate evaluation of low levels of E2 using electrochemical sensors through advanced modifications incorporating nanomaterials such as rGO-AuNPs/CNT on an SPE platform. The linear response of the sensor to E2 within the range of 0.05–1.00 µM E2 is illustrated in Figure 8A,B, with an R^2^ value of 0.9945 and a sample size (*n*) of seven. The limit of quantification (LOQ) was determined to be 0.66 µM, while the corresponding limit of detection (LOD) was calculated as low as 3 nM, indicating superior performance compared to previous studies on graphene-based materials such as RGO/CuTthP [27], CPE/GNR-FS-Au-CA [77], Gr–PANI [85], and GQDs-PSSA/GO [86]. Table 1 presents a comparison of the detection limits for estradiol using various graphene-based materials reported in the literature.

### 3.7. Reproducibility and Interference Studies

**Figure 9:**
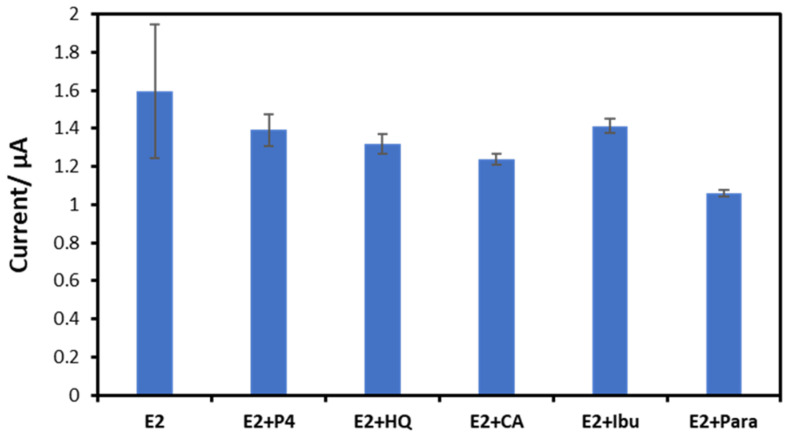
Effects of interferents that coexist in water on detecting estradiol. The initial concentration of target and interference (analyte:interferent) was 1:10 (µM/µM).

Stability and reproducibility studies were carried out to test the performance of the modified electrodes. In addition, reproducibility studies were carried out to test the rGO-AuNP-CNT/SPCE modified electrode using DPV measurements (*n* = 5) in the Britton–Robinson buffer. The results revealed that the fabricated sensor showed good reproducibility for a 5 µM E2 solution, with a coefficient of variance of 5.3%. To assess the influence of interferents, hydroquinone, dopamine, paracetamol, ibuprofen, and bisphenol A were investigated, as they are present in the water environment. An E2 concentration of 5 µM was used, with a 10-molar excess of the interferent under investigation as shown in Figure 9. No interference was observed for paracetamol and hydroquinone or for dopamine or ibuprofen. However, bisphenol A significantly influenced the oxidation peak current of estradiol, as it had a broad oxidation peak, increasing the signal for E2 by more than 50%, in contrast to less than 10% for the other interferents studied. 

### 3.8. Analysis of E2 in Drinking Water Samples

To ascertain the sensor’s analytical applicability for water testing, drinking water samples were fortified with E2 and investigated using the optimised SPE and voltammetric conditions. The samples (30 mL) were fortified with E2 at 0.5 and 0.9 µM with no dilution with buffer. Table 2 shows the recovery studies on water samples to determine the sensor’s performance, along with the spiked water samples with various amounts of E2.

## 4. Conclusions

This study has successfully demonstrated a one-step method for synthesising hybrid composites to modify screen-printed electrodes to determine E2 in water samples. The developed strategy, using a green synthesis of AuNPs and integration with reduced graphene oxide and carbon nanotubes, significantly improves the electrochemical characteristics and reproducibility of the sensor. 

The one-step method for synthesising hybrid composites for the modification of the SPE was developed by reducing AuNPs via a graphene oxide suspension with extracts obtained from bay laurel at room temperature. Correspondingly, combining them with carbon nanotubes produced rGO-AuNPs/CNT composite materials. The overall strategy for the green synthesis of AuNPs was developed using an organic, solvent-free method. In addition, the AuNP-decorated reduced graphene oxide serves as a dispersant for carbon nanotubes, removing the need to avoid the application of harsh solvents. Combining AuNP-integrated reduced graphene oxide and carbon nanotubes improved the performance of the sensor, showing superior electrochemical characteristics compared with other graphene-based sensors for E2 detection. In addition to reproducibility (relative standard of deviation of 2.58%), an LOD of 3 nM and an associated recovery of 92% were reported for the analysis of drinking water samples.

## Figures and Tables

**Table 1:** Literature report on electrochemical sensors for estradiol detection.

Electrode-Design-Modifier		Applied Technique	Sensor Type	Linear Range	Detection Limit	Reference
Glassy carbon electrode (GCE)	^2^ GR–PANI	^1^ DPV	Immunosensor	0.04–7.00 ng/mL	0.02 ng/mL	[85]
Glassy carbon electrode (GCE)	^3^ RGO/CuTthP	^1^ DPV	Electrochemical sensor	0.1–1.0 μM	5.3 nM	[27]
Carbon paste electrode	^4^ GNR-FS-Au-CA	^1^ DPV	Electrochemical sensor	0.1–5.0 μM	7.4 nM	[77]
Glassy carbon electrode (GCE)	^5^ GQDs-PSSA/GO	^1^ DPV	Electrochemical sensor	0.001–6.0 μM	0.23 nM	[86]
Screen-printed carbon electrode	^6^ rGO/AuNP/CNT)	^1^ DPV	Electrochemical sensor	0.05–1.0 μM	3 nM	This work

^1^ Differential pulse voltammetry (DPV). ^2^ Graphene–polyaniline (GR–PANI). ^3^ Reduced graphene oxide and a metal complex porphyrin (RGO/CuTthP). ^4^ Cysteamine self-assembled gold-nanoparticle-modified silica-decorated graphene-nanoribbon (GNR-FS-Au-CA). ^5^ Graphene quantum dots and poly (sulfosalicylic acid) (GQDs-PSSA/GO). ^6^ Gold nanoparticles (AuNPs) integrated on reduced graphene oxide–carbon nanotubes (rGO-AuNP/CNT).

**Table 2:** Determination for E2 in water samples using a rGO/AuNP/CNT/SPE sensor.

Added (μM)	Found (μM)	RSD	Recoveries (%)
0.5	0.46	2.7	92
0.5	0.45	2.5	90
0.9	0.96	3.2	106
0.9	0.92	2.8	102

## Data Availability

Not applicable.

## Supplementary Materials

The following supporting information can be downloaded at: https://www.mdpi.com/2079-6374/13/4/491/s1, Figure S1: Shows the peak currents in various supporting electrolytes; Figure S2: (A) Cyclic voltammograms (CVs) of bare SPE in 20 µM E2 at 25–170 mVs^−1^ scan rate. (B) Plot of current peak vs. scan rate. (C) Plot of current peak vs. square root of scan rate; Figure S3: Photograph of (A) Bay leaf extract (B) carbon-nanotubes in water (C) Gold nanoparticle reduced/graphene Oxide-Carbon nanotubes (D) Gold nanoparticle; Figure S4: (A) CV of Bare SPE immersed in 0.1 M KCl containing 5 mM [FeCN_6_]^−3/−4^ at 100 mVs^−1^ scan rate. (B) anodic current peak vs. square root of scan rate, and cathodic current peak vs. square root of scan rate of bare SPE. (C) CVs of rGO/CNT/SPE at different scan rates (25–200 mVs^−1^) 0.1 M KCl containing 5 mM [FeCN_6_]^−3/−4^. (D) Anodic current peak vs. square root of scan rate, and cathodic current peak vs. square root of scan rate of rGO/CNT/SPE electrode; Table S1: Electroactive surface area vs. electrode composition.
