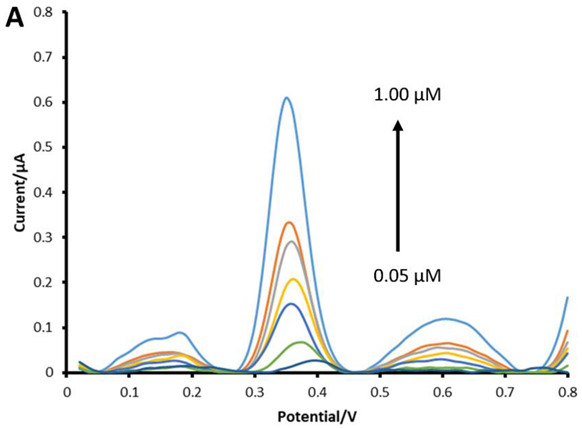# Correction: Musa et al. An Electrochemical Screen-Printed Sensor Based on Gold-Nanoparticle-Decorated Reduced Graphene Oxide–Carbon Nanotubes Composites for the Determination of 17-β Estradiol. *Biosensors* 2023, *13*, 491

**DOI:** 10.3390/bios13070756

**Published:** 2023-07-24

**Authors:** Auwal M. Musa, Janice Kiely, Richard Luxton, Kevin C. Honeychurch

**Affiliations:** 1 Institute of Bio-Sensing Technology (IBST), University of the West of England, Bristol BS16 1QY, UK; 2 Centre for Research in Biosciences (CRIB), School of Applied Sciences, University of the West of England, Bristol BS16 1QY, UK

## Error in Figure 8A

In the original publication [1], there is a mistake in Figure 8A as published. Figure 8A had an incorrect data range on the x-axis. The authors confirmed the mistake in Figure 8A’s x-axis, and a new Figure 8A, with a correct data range of 0.00 to 0.8V is now submitted. The corrected Figure 8A appears below. The authors apologize for any inconvenience caused and state that the scientific conclusions are unaffected. This correction was approved by the Academic Editor. The original publication has also been updated.