# Supplementary material for: An Electrochemical Screen-Printed Sensor Based on Gold-Nanoparticle-Decorated Reduced Graphene Oxide–Carbon Nanotubes Composites for the Determination of 17-β Estradiol
Source: Biosensors (Basel). 2023 Apr 19;13(4):491. doi: 10.3390/bios13040491 (PMC10136424; doi:10.3390/bios13040491)
Supplement: Supplementary file 1 [file biosensors-13-00491-s001.zip › biosensors-2313246-SI.pdf]

Supplementary Material

An Electrochemical Screen-Printed Sensor Based on Gold-Nanoparticle-Decorated Reduced Graphene Oxide–Carbon Nanotubes Composites for the Determination of 17-β Estradiol

Auwal M. Musa [1], Janice Kiely [2], Richard Luxton [2] and Kevin C. Honeychurch [1,2,*]

[1] Institute of Bio-Sensing Technology (IBST), University of the West of England, Bristol BS16 1QY, UK;
[2] Centre for Research in Biosciences (CRIB), School of Applied Sciences, University of the West of England, Bristol BS16 1QY, UK
* Correspondence: kevin.honeychurch@uwe.ac.uk; Tel.: +44-(0)1173287357

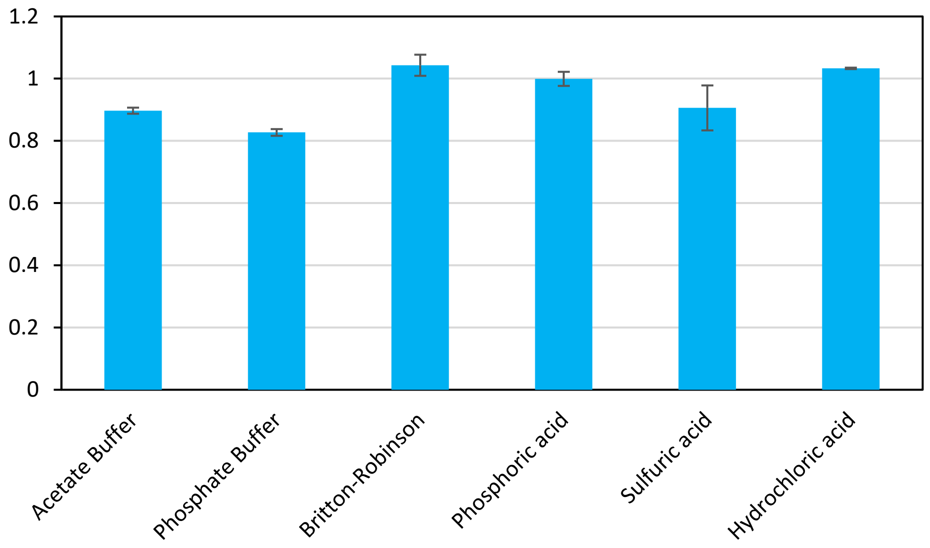

Figure. S1. Shows the peak currents in various supporting electrolytes.

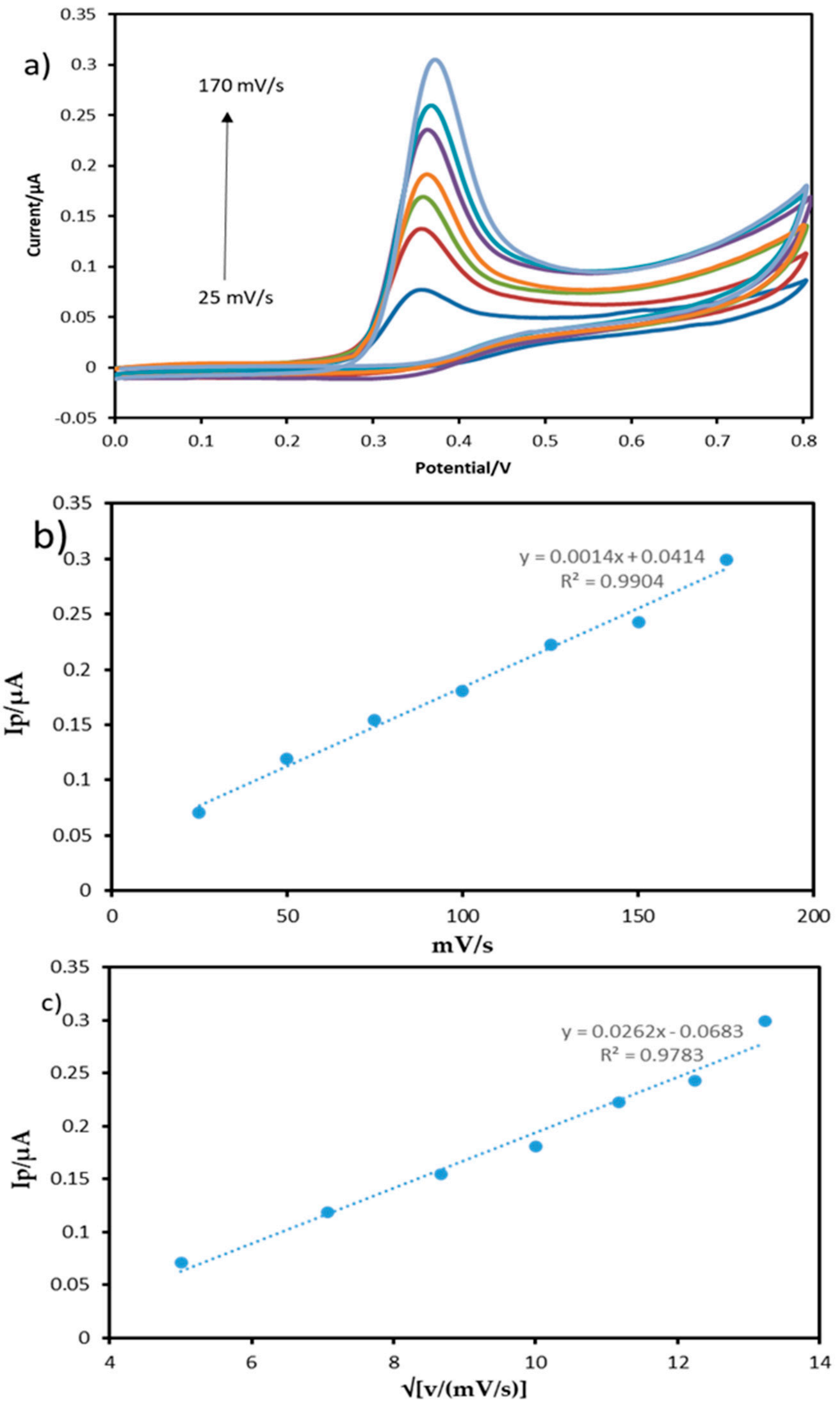

Figure S2. A) Cyclic voltammograms (CVs) of bare SPE in 20 μM E2 at 25-170 mVs^{-1} scan rate. b) Plot of current peak vs. scan rate. c) Plot of current peak vs. square root of scan rate.

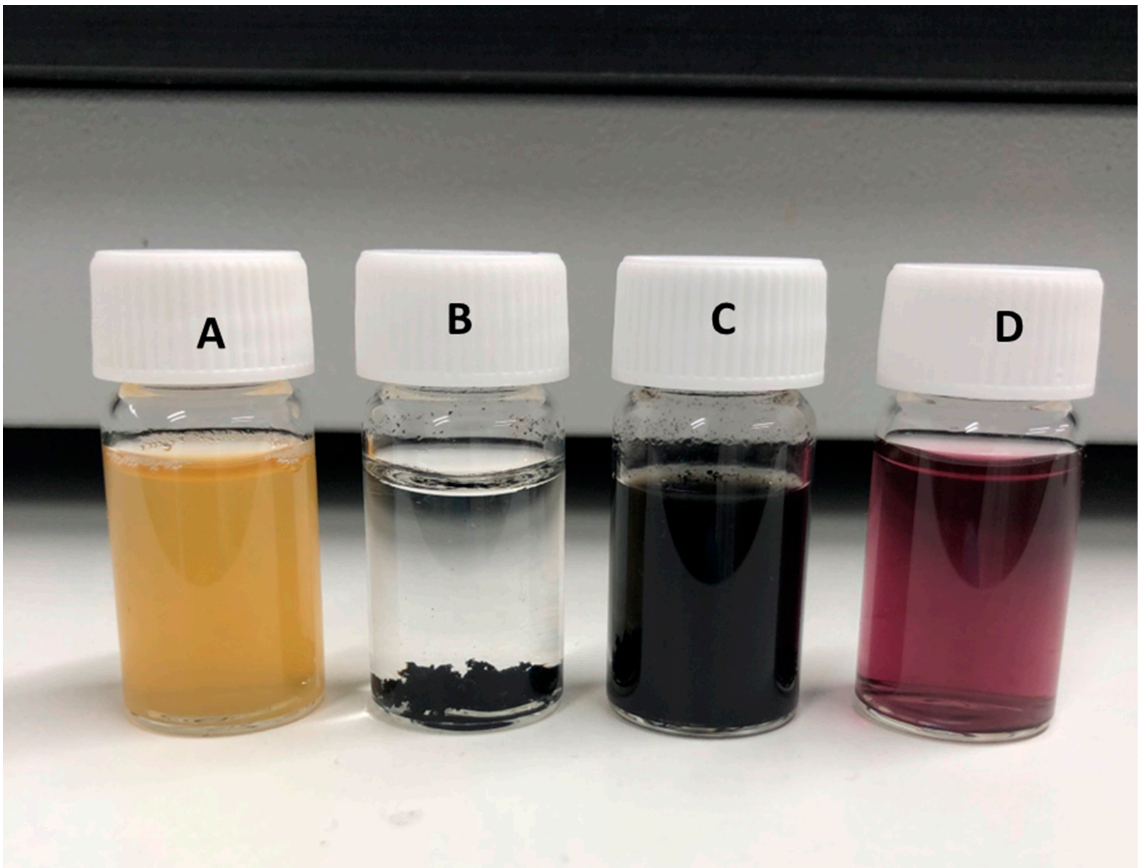

Figure S3. Photograph of (A) Bay leaf extract (B) carbon-nanotubes in water (C) Gold nanoparticle reduced/graphene Oxide-Carbon nanotubes D) Gold nanoparticle

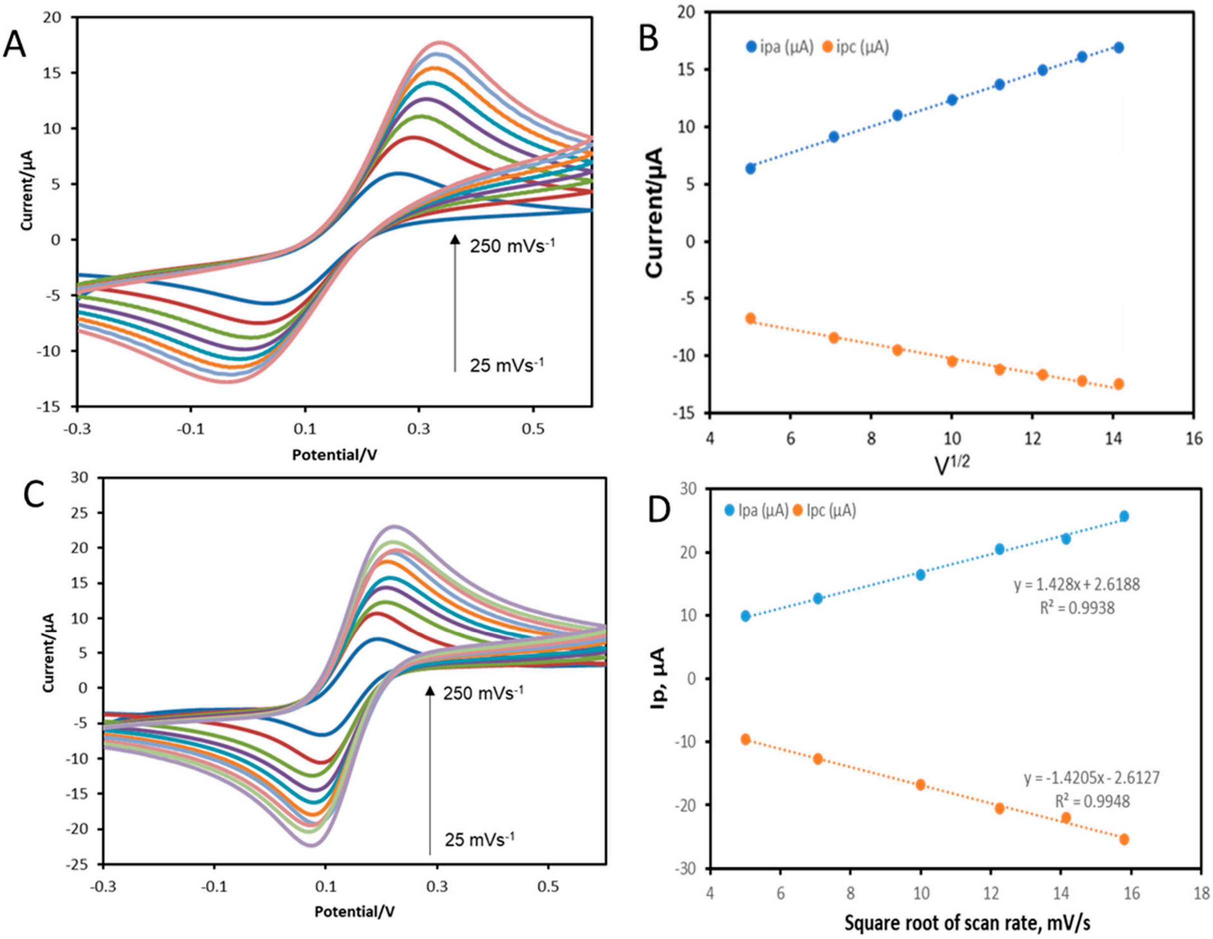

Figure S5 (A) CV of Bare SPE immersed in 0.1 M KCl containing 5 mM $[FeCN_6]^{-3/-4}$ at 100 mVs^{-1} scan rate. (B) anodic current peak vs. square root of scan rate, and cathodic current peak vs. square root of scan rate of bare SPE. (C) CVs of rGO/CNT/SPE at different scan rates (25-200 mVs^{-1}) 0.1 M KCl containing 5 mM $[FeCN_6]^{-3/-4}$. (D) Anodic current peak vs. square root of scan rate, and cathodic current peak vs. square root of scan rate of rGO/CNT/SPE electrode

Table S1. Electroactive surface area vs. electrode composition.

Electrode	Electroactive area
Bare SPE	0.009
GO-CNT	0.011
rGO-CNT	0.010
rGO-AuNP/CNT/SPE	0.014
rGO-AuNP	0.007

Abbreviations: Bare SPE: bare Screen printed electrode; GO-CNT: Graphene oxide-carbon nanotubes; rGO-CNT: Reduced graphene oxide-carbon nanotube; rGO-AuNP/CNT/SPE: gold nanoparticles-Reduced graphene oxide-carbon nanotube; rGO-AuNP: gold nanoparticles-Reduced graphene oxide

MatLab code

Baseline_magic

```
function [C,B] = baseline_magic(X,Y,N,M)
%-----------------------------------------------------------------------
%Function to take in x and y data from voltammetry scan, perform
%automatic baseline fitting and output baseline and baseline-corrected data
%--------------------
%AUTHOR: David Ferrier
%DATE: 01/11/19
%--------------------
%INPUTS:
%X - x-axis data (potential)
%Y - y-axis data (current)
```

```
%N - order of polynomial for fitting
%M - number of iterations
%--------------------
%OUTPUTS:
%C - baseline corrected data
%B - baseline
%------------------------------------------------------------------------
%------------------------------------------------------------------------
%Determine whether data is from a forward or reverse scan (positive =
%forward, negative = reverse)
direction = X(end,1)-X(1,1);
%----------------------------------
%----------------------------------
%Find baseline
Y_0 = Y;
for ind = 1:M
    P = polyfit(X,Y,N);          %perform least-squares fit to data
    F = fit_fill(P,X);
    Y = fit_shift(Y,F,direction);     %adjust data based on fit
end
B = F;
%----------------------------------
%----------------------------------
%Apply baseline correction to current data
C = correction(Y_0,F);
%----------------------------------
end
%------------------------------------------------------------------------
%SUBROUTINES
%------------------------------------------------------------------------
function F = fit_fill(P,X)
%----------------------------------
%Subroutine to create fit dataset based on least-sqaure fit parameters
```

```
%----------------------------------
L = length(X);
N = length(P) - 1;
F = zeros(L,1);
for ind = 1:L
    fu = 0;
    for dni = 1:N+1
        tvar = P(1,dni)*X(ind,1)^(N-(dni-1));
        fu = fu + tvar;
    end
    F(ind,1) = fu;
end
end
%-----------------------------------------------------------------------
%-----------------------------------------------------------------------
function F_prime = fit_shift(Y,F,direction)
%----------------------------------
%Subroutine to create adjusted dataset based on current iteration fit
%----------------------------------
L = length(Y);
F_prime = zeros(L,1);
if direction>0    %forward scan
    for ind = 1:L
        if Y(ind,1)>=F(ind,1)
            F_prime(ind,1) = F(ind,1);
        else
            F_prime(ind,1) = Y(ind,1);
        end
    end
    else                    %reverse scan

    for ind = 1:L
        if Y(ind,1)<=F(ind,1)
```

```
            F_prime(ind,1) = F(ind,1);
        else
            F_prime(ind,1) = Y(ind,1);
        end
    end

end
end
%-----------------------------------------------------------------------
%-----------------------------------------------------------------------
function Y_prime = correction(Y,F)
%----------------------------------
%Subroutine to create baseline corrected dataset
%----------------------------------
L = length(Y);
Y_prime = zeros(L,1);
for ind = 1:L
    Y_prime(ind,1) = Y(ind,1) - F(ind,1);
end
end
%-----------------------------------------------------------------------
%-----------------------------------------------------------------------
```

DPV_baseline

```
%-----------------------------------------------------------------------
%DPV_baseline
%Script to load DPV data and run baseline_magic
%-----------------------------------------------------------------------
%----------------------------------
%Load data file
```

```
input = 'Test_1';

fname = [input '.xlsx'];

dat = readmatrix(fname);

%--------------------------------------

%--------------------------------------

%Call baseline_magic for forward and reverse scans

N = 6;          %Order of polynomial for fitting purposes

M = 1000;    %Number of iterations

D = dat(3:end,:);

[C,B] = baseline_magic(D(:,1),D(:,2),N,M);

%--------------------------------------

%--------------------------------------

%Plot original data alongside baseline fit

figure

subplot(1,2,1);

plot(D(:,1),D(:,2),'b',D(:,1),B,'r')

title('DPV data')

xlabel('Potential/V')

ylabel('Current/\muA')

%-------------------

%Plot baseline corrected data

subplot(1,2,2);
```

```
plot(D(:,1),C,'b')

title('Baseline corrected data')

xlabel('Potential/V')

ylabel('Current/\muA')

%-------------------------------------

%-------------------------------------

%write output to file

output = zeros(length(D),4);

output(:,1) = D(:,1);

output(:,2) = D(:,2);

output(:,3) = B;

output(:,4) = C;

output_prime{1,1} = 'Potential (V)';

output_prime{1,2} = 'Current (uA)';

output_prime{1,3} = 'Baseline';

output_prime{1,4} = 'Corrected';

fnameout = [input '_BLcorrected' '.xlsx'];

writecell(output_prime,fnameout,'Sheet',1,'Range','A1')

writematrix(output,fnameout,'Sheet',1,'Range','A2')

%-------------------------------------

%-------------------------------------------------------------------

%-------------------------------------------------------------------
```
